# Hypertensive Emergency in Aortic Dissection and Thoracic Aortic Aneurysm—A Review of Management

**DOI:** 10.3390/ph2030066

**Published:** 2009-09-28

**Authors:** Prateek K. Gupta, Himani Gupta, Ali Khoynezhad

**Affiliations:** Division of Cardiothoracic and Vascular Surgery, Creighton University Medical Center, Omaha, NE 68131, USA

**Keywords:** hypertension, emergency, aortic, aneurysm, dissection

## Abstract

Over the last few decades, treatment for aortic dissection and thoracic aortic aneurysms has evolved significantly with improvement in outcomes. Treatment paradigms include medical, endovascular and surgical options. As aortic dissection presents as a hypertensive emergency, diligent control of BP is of utmost importance in order to reduce the progression of dissection with possible aortic branch malperfusion. Treatment should begin on arrival to the emergency department and continues in the intensive care unit, endovascular suite or the operating room. Novel antihypertensive medications with improved pharmacological profile and improved surgical techniques, have improved the prognosis of patients with aortic aneurysm and/or aortic dissection. Nevertheless, morbidity and mortality remain high and hypertensive emergency poses a significant challenge in aortic dissection and thoracic aortic aneurysms.

## Introduction

Hypertension is responsible for close to 7.1 million deaths each year [1]. Hypertensive crisis is a state in which systolic and diastolic blood pressure are acutely elevated to the point of causing target organ damage (TOD) [2] . It is estimated that 1% of patients with hypertension develop hypertensive crisis during the course of their life [3]. Treatment involves use of parenteral antihypertensive drugs for immediate reduction of blood pressure. Hypertensive emergency is often associated with aortic dissection, aneurysms and aortic surgeries. The choice of antihypertensive medication varies considerably due to lack of large prospective trials supporting the use of one therapeutic agent over another, as well as patient characteristics. The aim of this study was to review the surgical and medical therapy for hypertensive emergency associated with aortic dissection and aortic aneurysm.

## Aortic Dissection

Aortic dissection has been estimated to have an incidence of 3/100,000 per year [4,5]. Seventy percent of these patients are hypertensive and most of them are over 50 years of age, as there is reduced resistance of arterial walls with age [6,7,8]. Risk factors for aortic dissection include aortic coarctation, blunt trauma, connective tissue disorder, medial degeneration, bicuspid aortic valve, Marfan syndrome, Ehler-Danlos syndrome and pregnancy [4].

Patients frequently present with sudden onset chest pain radiating to the back. Pulse deficit occurs in 20% of patients with Type A dissection, while hypertension at initial presentation is more common in patients with Type B dissection [6,9]. Presentation, however, varies in practice, and diagnosis can remain elusive. Confirmation of aortic dissection is usually obtained by using contrast-enhanced computed tomography or transesophageal echocardiogram.

There are significant prognostic as well as treatment differences between patients with ascending dissection and those with descending dissection [8,10,11]. Up to 90% of untreated patients with acute dissection of the ascending aorta will die within three months [4]. The preferred treatment for all ascending aortic dissections is medical therapy along with emergent operation, unless there is a contraindication to operative intervention, while medical treatment is preferred for descending aortic dissections [12]. Operative intervention for descending aortic dissection is generally performed only for persistent pain, end organ ischemia due to malperfusion of aortic branch vessels, evidence of retrograde dissection to the ascending aorta, or chronic aneurysmal degeneration [8].

The immediate aims of operations for acute aortic dissection are to resect the damaged segment, excise the intimal tear, and obliterate the entry into the false lumen. The ultimate goals are to alleviate symptoms, reduce complications, and prevent rupture [4,8]. The operation is performed under cardiopulmonary bypass with or without hypothermic circulatory arrest. Dacron graft is used to replace the diseased segment. 

While outcomes of operative intervention for acute aortic dissection have improved over time, the morbidity and mortality are still significant. Over the last five years, most single center reports describe mortality of 5-25% [13,14,15,16,17,18]. Results however vary, with Mehta *et al*. for IRADs in 2002 reporting that among 547 patients with surgery for type A dissection, in-house mortality was 32.5% [19]. Predictors of death included age ≥70 years, abrupt onset of chest pain, hypotension, shock, or tamponade, kidney failure, pulse deficit, and abnormal electrocardiographic changes [19]. This highlights the fact that outcomes may be improved at high volume centers of excellence where protocols like the therapies described by the authors are more strictly followed. With type B dissections, Suzuki *et al*. reported an in-house mortality of 13% [20]. Predictors of in-hospital death with this type of dissection were absence of chest or back pain, hypotension or shock, and branch vessel ischemia of the iliac, mesenteric, and renal arteries [8,21].

## Aortic Aneurysm

The most common cause of perioperative death from aortic dissection is rupture into the pericardial or pleural cavity, or a coronary event. Late deaths, on the other hand, are most commonly due to the subsequent development and rupture of an aneurysm (a localized dilatation of the aorta with a diameter ≥50% greater than normal) [22,23]. DeBakey *et al*. in their retrospective analysis of the treatment of 527 patients with aortic dissection over a 20-year period, found that in Types I and IIIb, the incidences of aneurysm formation was 30% and 38%, respectively; in Type II it was 14%, and in Type IIIa 16%[22]. Crawford *et al*. showed that aortic aneurysm, if left untreated, leads to death from rupture or from associated disease within five years in 75% of cases [23]. According to Crawford *et al*., surgical treatment is recommended for new or enlarged aneurysms (5 to 6 cm in diameter), or if they produce symptoms [23]. They reported that in 4,170 patients with aneurysm of the aorta treated surgically, more than 30% had involvement of multiple aortic segments. Even in the late postoperative period, preoperative factors continue to exert an unfavorable influence on survival: The risk of perioperative death from aortic aneurysm was higher in those with preoperative angina or congestive heart failure. It is thus imperative that identification, monitoring, and treatment of patients for pre-existing cardiac conditions should be part of the treatment in early and late postoperative period [8,23].

## Management of Emergency Hypertension

Hypertensive crisis has been defined as systolic blood pressure (SBP) >180 mm Hg or diastolic blood pressure (DBP) >110 mm Hg [24]. Presence or absence of end-organ damage classifies it as hypertensive emergency or urgency. Hypertensive emergency calls for immediate reduction of blood pressure. It may be associated with aortic dissection, hypertensive encephalopathy, stroke, pulmonary edema, myocardial infarction, sympathetic crises and eclampsia.

In aortic dissection, target organ damage (TOD) occurs in the form of retrograde dissection into the heart, involvement of aortic branches, as well as endothelial injury. Untreated hypertensive emergency carries a 1-year mortality rate of 70% to 90%, and a 5-year mortality rate of nearly 100% [25]. Mortality is four times higher in patients with critical TOD [8,25]. With adequate control of blood pressure, the 1-year mortality and 5-year mortality rates decrease to 25% and 50%, respectively [26]. Oparil *et al*. recommend reduction of blood pressure within 1 hour of hospital presentation using parenteral antihypertensives in cases of hypertensive emergency, and within 1 day using oral antihypertensives in case of hypertensive urgency [25]. Some recommend reduction of SBP < 120 mm Hg within 20 minutes [27]. Others recommend reducing DBP to < 110 mm Hg within 5-10 minutes [7]. In individuals with chronic hypertension, due to ‘shift’ of autoregulatory range to the right, higher BP levels may be tolerated [7]. We have previously recommended reduction of SBP < 100 mm Hg or mean BP to <60 mm Hg within 20 minutes. Heart rate must be maintained between 55 and 65 beats/min [8]. 

The ideal antihypertensive agent in hypertensive emergency would preserves glomerular filtration rate and renal blood flow, have few or no drug interactions (especially anesthetics and vasoactive agents), have little or no potential for exacerbation of comorbid conditions (*e.g.*, congestive heart failure, chronic obstructive pulmonary disease), have rapid onset and offset of action, have minimal hypotension ("overshoot"), need minimal continuous blood pressure monitoring or frequent dose titration [25]. It should not have acute tolerance or tachyphylaxis to pharmacodynamic effect; be easy to use; should be convenient and safe; should not have toxic metabolites; have low cost, including total of drug and monitoring costs; have multiple formulations for short and long term use and have minimal sympathetic activation [25].

Antihypertensive therapy in acute aortic dissection aims specifically to lessen pulsatile load or aortic stress (dp/dt), in order to retard the propagation of the dissection and prevent aortic rupture [7]. The goals of treatment are to prevent myocardial ischemia, decrease left ventricular afterload, decrease myocardial oxygen consumption, and prevent rupture and bleeding from suture lines [8]. Few comparative studies or randomized, controlled trials exist that provide definitive conclusions and/or recommendations regarding the efficacy and safety of comparative agents.

### Nitroprusside

Nitroprusside is a potent direct arterial and venous dilator, acting through release of nitric oxide. It has a rapid onset of action, with a half-life of 3-4 minutes and thus is given in the form of continuous infusion. Dose is 0.3 µg/Kg/min IV infusion; with titration q2 min until desired response with maximum dose 10 µg/Kg/min. The hypotensive effects of nitroprusside can be unpredictable because it simultaneously causes potent venodilatation and peripheral arterial vasodilatation [25]. This is especially the case for patients with severe left ventricular hypertrophy and preload-dependent diastolic dysfunction. It has been shown to cause coronary steal; it can cause a significant reflex tachycardia, and it can decrease oxygen circulation. It is photosensitive, so it requires special handling [8]. Its most serious adverse effect is in the form of cyanide toxicity, which occurs due to accumulation of its metabolites thiocyanate/cyanide and its clinical presentation may vary leading to difficulty in diagnosis [7,28]. Thus, it is recommended that this drug be used only when other intravenous antihypertensive agents are not available [7].

### Nitroglycerin

Nitroglycerin acts by release of nitric oxide causing vasodilation, especially of the coronary arteries. It is primarily a venodilator, however, at higher doses it also causes arterial vasodilation [25]. It does not cause coronary steal. Initial dose is typically 5 µg/min IV infusion with increments by 5 µg every three to five minutes until desired response, with a maximum of 200 µg/min. A drawback is that it cannot be used for a prolonged duration as patients rapidly develop tolerance to it. Being predominantly a venodilator, it is subject to same hemodynamic issues such as nitroprusside. It may predispose to severe hypotension in patients with left ventricular hypertrophy and preload-dependent diastolic dysfunction. 

### Nicardipine

Nicardipine is a calcium channel blocker belonging to the dihydropyridine class. It causes cerebral and coronary vasodilation with only minimal negative inotropic effect, has minimal effects on atrioventricular nodal conduction, and has little effect on cardiac output or pulmonary arterial occlusion pressure [25,29]. In contrast to nitroprusside, the pharmacodynamic properties of nicardipine are favorable; however, their pharmacokinetic properties are unfavorable. Nicardipine has a very long half life; the β-half-life of nicardipine is approximately 40 min, whereas its γ-half-life is approximately 13 h. Because about 14% of the drug is eliminated during the γ-phase, the hypotensive effect can be prolonged [25]. In a study comparing the effect of nicardipine to nitroprusside in patients with severe postoperative hypertension, the two drugs had equivalent efficacy, but only nicardipine reduced both cardiac and cerebral ischemia [30]. Nicardipine is administered through a continuous infusion with starting dose at 5 mg/hr infusion. It is titrated by 2.5 mg/hr every 5 minutes with maximum of 15 mg/hr. In cardiac surgical patients, nicardipine has been shown to decrease arterial BP acutely with no effects on ventricular preload or cardiac output, suggesting that it has a minimal negative inotropic action [31]. With nicardipine, oxygen delivery to the cells is usually well maintained and oxygen requirements are unchanged [8,32]. Because nicardipine is metabolized primarily by the liver, it can be used in patients with renal insufficiency. However, there have been concerns with the use of calcium channel blockers in patients with coronary artery disease, possibly due to sympathetic activation, bleeding caused by inhibition of platelet aggregation, and proarrhythmic effects. There remains considerable debate about the role of calcium channel blockers as first line therapy [25,33,34,35].

### Clevidipine

Clevidipine is a new fourth-generation dihydropyridine L-type calcium channel blocker. It decreases blood pressure by direct arterial vasodilation with no effect on venous capacitance [29,36]. Compared to nicardipine, clevidipine has an ultra short half-life of 1 min, due to an easily hydrolysable ester group in its molecular structure, which gets rapidly metabolized by esterases in the blood [36,37]. It also has a faster onset of action, not needing hepatic biotransformation required by nicardipine. As clevidipine does not cause venodilatation like sodium nitroprusside and nitroglycerin, there is no preload reduction. This avoids reflex tachycardia, blood pressure lability and abrupt hypotension [38]. Initial IV infusion is at 0.4 mcg/kg/min or 1-2 mg/hr. The dose is doubled every 90 seconds up to 3.2 mcg/kg/min. Above that dose, increments are by 1.5 mcg/kg/min depending on patient’s response with a maximum of 8 mcg/kg/min.

The ECLIPSE trials compared clevidipine to nitroglycerin, sodium nitroprusside and nicardipine for management of hypertension in cardiac surgery patients [36]. They showed no difference in incidence of myocardial infarction, stroke or renal dysfunction. Mortality was significantly higher for patients treated with nitroprusside. Also, clevidipine was more effective in maintaining blood pressure within a specified range compared to nitroglycerin and nitroprusside. Fewer excursions beyond the BP range were seen with clevidipine, in comparison to nicardipine. Based on the rapid onset and offset of action, and relatively safe pharmacological profile, clevidipine should be considered one of the first line drugs for management of acute hypertensive emergency.

### Fenoldopam

Fenoldopam is a selective dopamine-1 agonist (the only drug in its class) and selective arteriolar/renal dilator. It has a rapid onset of action and a short duration of action, with a half-life of five minutes. There is a very clear dose-response relationship to blood pressure reduction. It is administered as a constant infusion at dosages of 0.01 to 1.6 mcg/kg/min, producing steady-state plasma concentrations that are proportional to infusion rates. Blood pressure reduction is dose-dependent. The onset of blood pressure response is rapid, with the 15-min response (three half-lives) representing 50% to 100% of the 1-hour response. It is therefore recommended that the drug be titrated no more frequently than every 15 min (and less frequently as goal pressure is approached) [25].

It does not produce coronary steal. Because it improves creatinine clearance, urine flow rates, and sodium excretion in severely hypotensive patients with impaired renal function, fenoldopam may be the drug of choice in these patients [39]. Compared to nitroprusside, fenoldopam has a longer half-life and does not cause venodilatation, thus blood pressure reduction is more predictable. Fenoldopam is not metabolized by cytochrome P-450, and has no known major drug interactions. Furthermore, its pharmacokinetics is not altered in the presence of hepatic or renal insufficiency [25]. Like other vasodilators, fenoldopam may cause reflex tachycardia and nonspecific T-wave and ST changes on the ECG. It may reduce serum potassium, and it produces a mild tolerance after long-term infusion [8,25].

### β-Blockers

In aortic dissection and aortic aneurysm, propagation of aortic dissection depends not only on the absolute blood pressure, but also on the velocity of left ventricular contraction [7]. A vasodilator alone, instead of decreasing the heart rate, may even cause reflex tachycardia, thus causing propagation of the dissection. Therefore, the optimum treatment involves a combination of a parenteral *β* blocker and a vasodilator, with heart rate targeted around 55 to 65 beats/minutes [8]. The *β* blocker of choice in this situation is generally esmolol and alternatively, labetalol or metoprolol [7].

Esmolol is a *β*1 antagonist while labetalol is a combined *α*1, *β*1 and *β*2 antagonist with an alpha to beta blocking ratio of 1:7 [40]. Both of them, by slowing down the heart rate, also reduce the myocardial oxygen demand. Esmolol reduces blood pressure by reducing cardiac output and inhibiting renin release, while labetalol decreases afterload directly and also inhibits renin release [25]. Their disadvantage is in the form of their negative inotropic effect, and possible reaction in patients with reactive airway disease. Half life of esmolol is around nine minutes, while that of labetalol is 5.5 hours [41].

The hypotensive effect of labetalol begins within 2–5 minutes after its intravenous administration, reaching a peak at 5–15 minutes following administration and lasting for about 2–4 hours [42,43]. Labetalol may be administered as a loading dose of 20 mg, followed by repeated incremental doses of 20–80 mg at 10-minute intervals until the desired BP is achieved. Alternatively, after the initial loading dose an infusion commencing at 1–2 mg/min and titrated up to until the desired hypotensive effect is achieved is particularly effective. Bolus injections of 1–2 mg/kg have been reported to produce precipitous falls in BP and should therefore be avoided [41,44].

**Approach to acute aortic dissections (AAD) in the emergency department**

1) Have a high index of suspicion for AAD
  a) History:
    i) Sudden onset, severe, sharp or tearing back pain, chest pain, shoulder pain, or abdominal pain
    - ii) Older than 60 years, history of hypertension, aortic dissection or aortic aneurysm (of family history of such), previous cardiac surgery, connective tissue disorder (Bicuspid aortic valve, Marfan syndrome, Ehler-Danlos syndrome, Loeys-Dietz syndrome), or peripartum
  b) Physical examination:
    (1) Pulse deficit, blood pressure differential in various extremities
    (2) Neurological deficits
    (3) Abdominal pain, flank pain
2) General measures:
  a) Establish two large bore (18gauge) IV’s
  b) Administer supplemental oxygen by nasal cannula or nonrebreather mask
  c) Put patient on cardiac monitor
  d) Get an EKG, portable chest X-ray, place a Foley catheter
  e) Obtain CBC, chemistry panel, coagulation panel, UA, CK, Troponin, d-dimer
  f) Type and cross 10 units packed red blood cells (PRBC’s)
  g) Set up an arterial line
3) Early cardiothoracic surgical consultation
4) Definitive imaging:
  a) Computed tomography angiogram (CTA)
  b) Transesophageal echocardiogram
  c) Magnetic resonance angiogram (MRA)
  d) Intravascular ultrasound
  e) Aortography
5) Blood pressure, heart rate, and pain management
  a) First line: β-blockers
    i) Labetalol, bolus (15 mg) ± a drip (5 mg/hour),
  b) If hypertension persists, add:
    i) Nicardipine drip (starting dose: 5 mg/h)
  c) If tachycardia persists, add:
    i) Esmolol (loading 0.5 mg/kg over 2–5 min, followed by a drip of 10–20 μg/kg/min)
    - ii) Diltiazem drip (loading 0.25 mg/kg over 2–5 min, followed by a drip of 5mg/h)
  d) Goals:
    (1) Heart rate _60 beats/min
    (2) Systolic blood pressure _100 mm Hg
  e) Morphine (for pain relief)
6) Hemodynamically unstable patients
  a) Tracheal intubation, mechanical ventilation
  b) Blood pressure support with crystalloid and colloid (PRBC’s if rupture is suspected)
  c) TEE at bedside in the Emergency Department or in the OR
  d) Pericardiocentesis is not recommended (class III)

The onset of action of esmolol is within 60 seconds, with duration of action of 10–20 minutes. However, because it is metabolized by red blood cell (RBC) esterases, any condition that precipitates anemia will prolong its ‘short half-life’. The metabolism of esmolol is via rapid hydrolysis of ester linkages by RBC esterases and is not dependant on renal or hepatic function. Typically, the drug is administered as a 500–1000 μg/kg loading dose over 1 minute, followed by an infusion starting at 50 μg/kg/min and increasing up to 300 μg/kg/min as necessary [41,45,46]

## Conclusions

Hypertensive emergency with aortic dissection and symptomatic aortic aneurysm is associated with major morbidity and mortality. Poorly controlled hypertension may cause aortic disruption with potentially fatal bleeding, as well as progression of the intimal flap in case of aortic dissection [8]. It thus becomes imperative to tightly control blood pressure using above mentioned parenteral antihypertensives. A combination of a vasodilator and *β-*blocker is preferred. A short-acting dihydropyridine calcium channel blocker may be used along with esmolol, labetalol or metoprolol. The dosage titration of the *β-*blocker is very slow compared with nicardipine or clevidipine. Once oral intake is established, parenteral antihypertensives are slowly switched to oral, keeping the blood pressure and heart rate tightly regulated at all times. 

With the increase in life expectancy and prevalence of arterial hypertension, the incidence of aortic dissection and aortic aneurysm has risen. Since the earliest aortic resections in 1950s, when likelihood of surviving surgery for aortic dissection for one year was only 50%, refinements in surgical techniques, novel anti-impulsive medications, better graft materials, improved cardiopulmonary-bypass circuits, and better selection of patients for surgical procedures have greatly increased survival [8,27,47,48]. Nevertheless, according to the International Registry of Acute Aortic Dissection (IRAD) morbidity and mortality still remain high and hypertensive emergency poses a significant challenge in aortic dissection and thoracic aortic aneurysms [6].

## References

[1] Brundtland G.H. (2002). From the World Health Organization. Reducing risks to health, promoting healthy life. JAMA.

[2] Murphy C. (1995). Hypertensive emergencies. Emerg. Med. Clin. North Am..

[3] McRae R.P., Liebson P.R. (1986). Hypertensive crisis. Med. Clin. North Am..

[4] Kouchoukos N.T., Dougenis D. (1997). Surgery of the thoracic aorta. N. Engl. J. Med..

[5] Meszaros I., Morocz J., Szlavi J., Schmidt J., Tornoci L., Nagy L., Szep L. (2000). Epidemiology and clinicopathology of aortic dissection. Chest.

[6] Hagan P.G., Nienaber C.A., Isselbacher E.M., Bruckman D., Karavite D.J., Russman P.L., Evangelista A., Fattori R., Suzuki T., Oh J.K., Moore A.G., Malouf J.F., Pape L.A., Gaca C., Sechtem U., Lenferink S., Deutsch H.J., Diedrichs H., Robles J., Llovet A., Gilon D., Das S.K., Armstrong W.F., Deeb G.M., Eagle K.A. (2000). The International Registry of Acute Aortic Dissection (IRAD): New insights into an old disease. JAMA.

[7] Varon J., Marik P.E. (2000). The diagnosis and management of hypertensive crises. Chest.

[8] Khoynezhad A., Plestis K.A. (2006). Managing emergency hypertension in aortic dissection and aortic aneurysm surgery. J. Card Surg..

[9] Erbel R., Alfonso F., Boileau C., Dirsch O., Eber B., Haverich A., Rakowski H., Struyven J., Radegran K., Sechtem U., Taylor J., Zollikofer C., Klein W.W., Mulder B., Providencia L.A. (2001). Diagnosis and management of aortic dissection. Eur. Heart J..

[10] Daily P.O., Trueblood H.W., Stinson E.B., Wuerflein R.D., Shumway N.E. (1970). Management of acute aortic dissections. Ann. Thorac. Surg..

[11] Tran P., Khoynezhad A. (2009). Current medical management of type B aortic dissection. Ther. Clin. Risk Manage..

[12] Khoynezhad A. (2007). Antihypertensive therapy in a patient with type B and de novo type A aortic dissection: case study. Crit. Care Clin..

[13] Pompilio G., Spirito R., Alamanni F., Agrifoglio M., Polvani G., Porqueddu M., Reali M., Biglioli P. (2001). Determinants of early and late outcome after surgery for type A aortic dissection. World J. Surg..

[14] Stevens L.M., Madsen J.C., Isselbacher E.M., Khairy P., Macgillivray T.E., Hilgenberg A.D., Agnihotri A.K. (2009). Surgical management and long-term outcomes for acute ascending aortic dissection. J. Thorac. Cardiovasc. Surg..

[15] Santini F., Montalbano G., Casali G., Messina A., Iafrancesco M., Luciani G.B., Rossi A., Mazzucco A. (2007). Clinical presentation is the main predictor of in-hospital death for patients with acute type A aortic dissection admitted for surgical treatment: a 25 years experience. Int. J. Cardiol..

[16] Kazui T., Washiyama N., Bashar A.H., Terada H., Suzuki T., Ohkura K., Yamashita K. (2002). Surgical outcome of acute type A aortic dissection: analysis of risk factors. Ann. Thorac. Surg..

[17] Ehrlich M.P., Ergin M.A., McCullough J.N., Lansman S.L., Galla J.D., Bodian C.A., Apaydin A., Griepp R.B. (2000). Results of immediate surgical treatment of all acute type A dissections. Circulation.

[18] Suehiro K., Pritzwald-Stegmann P., West T., Kerr A.R., Haydock D.A. (2006). Surgery for acute type a aortic dissection a 37-year experience in Green Lane Hospital. Heart Lung Circ..

[19] Mehta R.H., Suzuki T., Hagan P.G., Bossone E., Gilon D., Llovet A., Maroto L.C., Cooper J.V., Smith D.E., Armstrong W.F., Nienaber C.A., Eagle K.A. (2002). Predicting death in patients with acute type a aortic dissection. Circulation.

[20] Suzuki T., Mehta R.H., Ince H., Nagai R., Sakomura Y., Weber F., Sumiyoshi T., Bossone E., Trimarchi S., Cooper J.V., Smith D.E., Isselbacher E.M., Eagle K.A., Nienaber C.A. (2003). Clinical profiles and outcomes of acute type B aortic dissection in the current era: lessons from the International Registry of Aortic Dissection (IRAD). Circulation.

[21] Prisant L.M., Nalamolu V.R. (2005). Aortic dissection. J. Clin. Hypertens. (Greenwich.).

[22] DeBakey M.E., McCollum C.H., Crawford E.S., Morris G.C., Howell J., Noon G.P., Lawrie G. (1982). Dissection and dissecting aneurysms of the aorta: twenty-year follow-up of five hundred twenty-seven patients treated surgically. Surgery.

[23] Crawford E.S., Coselli J.S., Svensson L.G., Safi H.J., Hess K.R. (1990). Diffuse aneurysmal disease (chronic aortic dissection, Marfan, and mega aorta syndromes) and multiple aneurys. Treatment by subtotal and total aortic replacement emphasizing the elephant trunk operation. Ann. Surg..

[24] Marik P.E., Varon J. (2007). Hypertensive crises: challenges and management. Chest.

[25] Oparil S., Aronson S., Deeb G.M., Epstein M., Levy J.H., Luther R.R., Prielipp R., Taylor A. (1999). Fenoldopam: a new parenteral antihypertensive: consensus roundtable on the management of perioperative hypertension and hypertensive crises. Am. J. Hypertens..

[26] Webster J., Petrie J.C., Jeffers T.A., Lovell H.G. (1993). Accelerated hypertension--patterns of mortality and clinical factors affecting outcome in treated patients. Q. J. Med..

[27] Elliott W.J. (2004). Clinical features and management of selected hypertensive emergencies. J. Clin. Hypertens. (Greenwich.).

[28] Vidt D.G. (2004). Hypertensive crises: emergencies and urgencies. J. Clin. Hypertens. (Greenwich.).

[29] Khoynezhad A., Dobesh P.P., Stacy Z., Jalali Z. (2008). The role of intravenous dihydropyridine calcium channel blockers in the perioperative management of patients undergoing coronary artery bypass surgery. Curr. Vasc. Pharmacol..

[30] Halpern N.A., Sladen R.N., Goldberg J.S., Neely C., Wood M., Alicea M., Krakoff L.R., Greenstein R. (1990). Nicardipine infusion for postoperative hypertension after surgery of the head and neck. Crit Care Med..

[31] Cheung A.T., Guvakov D.V., Weiss S.J., Savino J.S., Salgo I.S., Meng Q.C. (1999). Nicardipine intravenous bolus dosing for acutely decreasing arterial blood pressure during general anesthesia for cardiac operations: pharmacokinetics, pharmacodynamics, and associated effects on left ventricular function. Anesth. Analg..

[32] Vincent J.L., Berlot G., Preiser J.C., Engelman E., Dereume J.P., Khan R.J. (1997). Intravenous nicardipine in the treatment of postoperative arterial hypertension. J. Cardiothorac. Vasc. Anesth..

[33] Epstein M. (1996). Calcium antagonists: still appropriate as first line antihypertensive agents. Am. J. Hypertens..

[34] Furberg C.D., Psaty B.M., Meyer J.V. (1995). Nifedipine. Dose-related increase in mortality in patients with coronary heart disease. Circulation.

[35] Legault C., Furberg C.D., Wagenknecht L.E., Rogers A.T., Stump D.A., Coker L., Troost B.T., Hammon J.W. (1996). Nimodipine neuroprotection in cardiac valve replacement: report of an early terminated trial. Stroke.

[36] Aronson S., Dyke C.M., Stierer K.A., Levy J.H., Cheung A.T., Lumb P.D., Kereiakes D.J., Newman M.F. (2008). The ECLIPSE trials: comparative studies of clevidipine to nitroglycerin, sodium nitroprusside, and nicardipine for acute hypertension treatment in cardiac surgery patients. Anesth. Analg..

[37] Nordlander M., Sjoquist P.O., Ericsson H., Ryden L. (2004). Pharmacodynamic, pharmacokinetic and clinical effects of clevidipine, an ultrashort-acting calcium antagonist for rapid blood pressure control. Cardiovasc. Drug Rev..

[38] Singla N., Warltier D.C., Gandhi S.D., Lumb P.D., Sladen R.N., Aronson S., Newman M.F., Corwin H.L. (2008). Treatment of acute postoperative hypertension in cardiac surgery patients: an efficacy study of clevidipine assessing its postoperative antihypertensive effect in cardiac surgery-2 (ESCAPE-2), a randomized, double-blind, placebo-controlled trial. Anesth. Analg..

[39] Shusterman N.H., Elliott W.J., White W.B. (1993). Fenoldopam, but not nitroprusside, improves renal function in severely hypertensive patients with impaired renal function. Am. J. Med..

[40] Lund-Johansen P. (1984). Pharmacology of combined alpha-beta-blockade. II. Haemodynamic effects of labetalol. Drugs.

[41] Varon J. (2008). Treatment of acute severe hypertension: current and newer agents. Drugs.

[42] Kanto J., Allonen H., Kleimola T., Mantyla R. (1981). Pharmacokinetics of labetalol in healthy volunteers. Int. J. Clin. Pharmacol. Ther. Toxicol..

[43] Goldberg M.E., Clark S., Joseph J., Moritz H., Maguire D., Seltzer J.L., Turlapaty P. (1990). Nicardipine versus placebo for the treatment of postoperative hypertension. Am. Heart J..

[44] Rosei E.A., Trust P.M., Brown J.J., Lever A.F., Robertson J.I. (1975). Letter: Intravenous labetalol in severe hypertension. Lancet.

[45] Lowenthal D.T., Porter R.S., Saris S.D., Bies C.M., Slegowski M.B., Staudacher A. (1985). Clinical pharmacology, pharmacodynamics and interactions with esmolol. Am. J. Cardiol..

[46] Gray R.J. (1988). Managing critically ill patients with esmolol. An ultra short-acting beta-adrenergic blocker. Chest.

[47] Cooley D.A., De Bakey M.E. (1956). Resection of entire ascending aorta in fusiform aneurysm using cardiac bypass. J. Am. Med. Assoc..

[48] De Bakey M.E., Crawford E.S., Cooley D.A., Morris G.C. (1957). Successful resection of fusiform aneurysm of aortic arch with replacement by homograft. Surg. Gynecol. Obstet..

